# Efficient Fall Detection from Wrist-Worn IMU Signals via Knowledge Distillation: A Lightweight CNN Approach Using the UMAFall Dataset

**DOI:** 10.3390/s26113328

**Published:** 2026-05-24

**Authors:** Ali Taheri, Mina Salehi, Jeong Ho Kim

**Affiliations:** 1 Department of Electrical and Computer Engineering, Texas A&M University, College Station, TX 77843, USA; ali.taheri@tamu.edu; 2 School of Nutrition and Public Health, College of Health, Oregon State University, Corvallis, OR 97331, USA; salehim@oregonstate.edu; 3 Department of Environmental and Occupational Health, School of Public Health, Texas A&M University, College Station, TX 77843, USA

**Keywords:** fall detection, wearable sensors, wrist-worn IMU, knowledge distillation, lightweight CNN, UMAFall

## Abstract

Falls are a major contributor to morbidity and mortality among older adults, and timely fall detection can help reduce the severity of fall-related outcomes. Wearable inertial measurement unit (IMU) sensors offer a promising solution for fall detection; however, many existing approaches rely on multiple sensing locations and computationally intensive models, which can limit their practicality for resource-constrained wearable devices. This study proposes a knowledge distillation framework for efficient wrist-based fall detection using the publicly available University of Málaga fall detection dataset (UMAFall), a benchmark dataset for human activity recognition and fall detection. Although UMAFall was not collected from older adults, it provides a useful public benchmark for evaluating IMU-based fall detection methods. Knowledge distillation was implemented using a teacher–student framework, in which a high-capacity teacher model trained with IMU data from four body locations (waist, wrist, ankle, and chest) provided soft targets for guiding a compact wrist-only CNN student model. In a held-out test evaluation using Subjects 2 and 5, the teacher model achieved 97.6% accuracy and an F1 score of 96.7%, with approximately 1.3 million trainable parameters. The independently trained wrist-based CNN achieved 90.2% accuracy and an F1 score of 87.1%. After applying knowledge distillation, the student model improved to 95.1% accuracy and an F1 score of 93.3% while maintaining the same lightweight architecture. A supplementary leave-one-subject-out analysis showed slightly higher and more stable AUC for KD-CNN than the independently trained CNN (0.96 ± 0.03 vs. 0.94 ± 0.07). These findings suggest that knowledge distillation can improve wrist-only fall detection in this feasibility evaluation, but further validation using older adults and real-world smartwatch data is needed.

## 1. Introduction

Falls are a leading cause of injury, permanent disability, and fatality among adults aged 65 years and older [1]. In the U.S., over 14 million older adults (about 1 in 4) experience falls each year [2]. Approximately 37% of these falls result in serious injuries that restrict physical activity or require medical attention [1]. Severe fall injuries can lead to long-term disability, reduced mobility, psychological distress, diminished quality of life, and even death [3,4,5]. Beyond their clinical impact, falls impose substantial health and economic burdens. Based on the World Health Organization (WHO) report, approximately 37 million annual falls are severe enough to require some kind of medical treatment [6]. In the U.S., the medical cost of falls among the elderly was estimated at $50 billion in 2015 [7]. Moreover, with the rapid aging of the global population, which results in declined mobility, balance, and reaction time, the number of individuals vulnerable to age-related falls is increasing globally [8,9,10]. Consequently, the burden of fall-related injuries is expected to rise substantially, highlighting the need for effective strategies to assess and mitigate the risk of falls and associated outcomes among older adults.

Fall detection systems have been developed to enable timely recognition of fall events and facilitate rapid emergency responses, especially among older adults. Timely fall detection not only facilitates prompt medical intervention and reduces the injury severity and mortality but also supports older adults in maintaining independence by providing safety and reassurance within their homes [11,12]. Therefore, automated fall detection systems have become a vital component of all fall prevention and management strategies [13]. These systems are designed to automatically identify fall events and notify caregivers or emergency services, ensuring timely assistance for older individuals living alone or in care facilities [12,14]. Given the high prevalence and serious consequences of falls among older adults, substantial research efforts over the past decades have focused on developing reliable, accurate, and minimally intrusive fall detection systems.

Fall detection technologies can be broadly classified into two main categories: computer vision-based systems and wearable sensor-based systems. Vision-based fall detection systems leverage deep learning algorithms to analyze visual inputs, monitor human activities, extract key features such as posture or skeletal movements, and automatically identify fall events in video sequences [15]. This approach allows simultaneous monitoring of multiple individuals within a given environment without causing discomfort or inconvenience associated with wearable devices, making it especially suitable for elderly populations [16]. Despite these advantages, vision-based detection systems face significant challenges, including privacy concerns, high computational demands, sensitivity to lighting conditions, and susceptibility to occlusion [17]. Consequently, while these systems have shown promising performance under controlled conditions, further research is necessary to establish their accuracy, feasibility, and effectiveness in real-world settings.

Wearable sensor-based fall detection systems offer an effective alternative for addressing some of the limitations inherent to vision-based methods. These systems employ various sensors integrated into wearable devices, often placed on different body locations, to continuously monitor and analyze human movements for detecting falls [16]. The most commonly used wearable sensors in fall detection include accelerometers and gyroscopes, which are favored due to their reliability, compact size, and low power consumption [18,19]. Accelerometers measure linear acceleration, providing critical data on sudden changes in speed and orientation, while gyroscopes capture angular velocity, aiding in identifying rotational movements characteristic of falls [11].

Inertial measurement units (IMUs), which combine accelerometers and gyroscopes into a single integrated device, have gained increasing popularity for fall detection in recent years. IMUs enhance the accuracy of fall detection by offering comprehensive movement data that effectively discriminates between falls and activities of daily living [11,20,21]. Unlike vision-based approaches, wearable sensors, such as IMUs, inherently mitigate privacy concerns, reduce computational demands, and are less sensitive to environmental factors such as lighting and occlusions, which make them particularly suitable for real-world applications and for effective fall detection among elderly populations [22,23]. Recent research has focused on the feasibility of single-sensor IMU systems for fall detection, aiming to balance detection accuracy with user comfort and acceptance. In this context, sensor location is a critical factor influencing the effectiveness of fall detection systems [19,24].

The trunk is often considered the optimal location for fall detection because of its proximity to the body’s center of mass (CoM). This position enables the more reliable capture of whole-body acceleration patterns associated with falls while reducing confounding signals from sudden, non-fall-related limb movements [25,26,27]. However, trunk-mounted IMU sensors may cause discomfort and inconvenience for users, which may reduce older adults’ willingness to wear the device for extended periods [28,29]. Compared to trunk-based systems, wrist-worn devices are less intrusive and more user-friendly, especially when integrated into everyday accessories like watches and bracelets [30,31]. Nevertheless, the wrist has been greatly overlooked for fall detection applications because of the hand’s frequent and rapid movements during activities of daily living (ADLs), resulting in a high number of false-positive alarms [32].

Recent advances in deep learning have significantly improved the effectiveness of wearable IMU-based fall detection systems, especially those worn on the wrist. Deep neural architectures, including convolutional neural networks (CNNs), long short-term memory (LSTM) networks, hybrid CNN-LSTM models, and Transformer-based approaches, have demonstrated high detection accuracy when applied to inertial data, including wrist-worn sensors [29,33,34,35]. These studies have shown promising results with detection accuracies exceeding 95%. However, these models often require substantial computational resources, which limit their deployment on wearable devices with constrained processing power, memory, and battery capacity. To address this limitation, knowledge distillation (KD) has emerged as an effective strategy for transferring knowledge from a large, high-capacity teacher model to a smaller, computationally efficient student model while preserving detection performance [36]. For example, knowledge-distillation-based fall detection frameworks have shown that lightweight student models can achieve comparable accuracy while significantly reducing computational cost, thereby improving their suitability for embedded and wearable applications [37,38].

Despite these advances, existing knowledge-distillation-based fall detection studies have primarily focused on reducing model complexity while maintaining performance, and they have generally assumed that the student model uses the same sensing configuration as the teacher model. Recent work on wearable human activity recognition has demonstrated that knowledge distillation can also enable efficient models that use fewer sensing modalities or sensor locations by transferring knowledge from a multimodal teacher model with several sensing locations to a lightweight student model operating with reduced sensing points [39]. This approach is particularly useful for wrist-based fall detection, where reducing the number of sensors can improve user comfort and long-term use but may compromise detection accuracy due to the lack of sufficient information for reliably detecting falls. Therefore, the potential of knowledge distillation as a solution not only to reduce the complexity of fall detection models but also to enable reliable detection from wrist-worn IMU sensors remains largely unknown.

Although the broader motivation of this work is fall detection for older adults, the present study was designed as a methodological feasibility evaluation rather than a direct validation in the target population. The proposed teacher–student framework requires IMU data collected simultaneously from multiple body locations to train the teacher model while also including wrist-worn IMU signals to evaluate the reduced-sensor student model. However, as summarized in Table 1, most publicly available wearable fall-detection datasets include only one or two sensing locations, limiting their suitability for evaluating knowledge transfer from a multi-location teacher model to a wrist-only student model. Therefore, a dataset containing both multi-location and wrist-worn IMU data was needed to examine whether information learned from multiple body locations could improve wrist-based fall detection.

Based on these requirements, this study used the UMAFall dataset, a publicly available benchmark developed by the University of Málaga for human activity recognition and fall detection [41]. UMAFall provides synchronized IMU data from several body locations, including the waist, wrist, ankle, and chest, making it well suited for evaluating the proposed teacher–student framework. Because the dataset was not collected from older adults, the findings should be interpreted as evidence of methodological feasibility rather than direct evidence of performance in the intended older-adult population.

Using this dataset, the present study developed a wrist-based fall-detection framework based on teacher–student knowledge distillation. The main contribution is the development of a lightweight CNN-based student model that operates solely on wrist-worn IMU signals, while its training is guided by a high-capacity Transformer-based teacher model trained using multi-location IMU inputs. Consistent with knowledge distillation theory, the teacher model provides softened output probabilities that contain more informative class-relationship patterns than hard labels alone. By learning from these soft targets, the wrist-only student model may capture fall-related information that would be difficult to learn from wrist data alone. Accordingly, the proposed framework aims to reduce both computational complexity and the number of required sensing locations while maintaining acceptable fall-detection performance, thereby supporting the development of more practical wearable fall-detection systems.

## 2. Materials and Methods

### 2.1. Publicly Available Dataset

Several public datasets have been developed for wearable sensor-based fall detection and include different combinations of activities, fall types, sensor modalities, and sensing locations. Table 1 summarizes 11 publicly available datasets reported in [40]. For the present study, dataset selection was guided by the requirements of the proposed teacher–student framework, which required synchronized multi-location IMU data for teacher-model training and wrist-worn IMU data for student-model evaluation. Among the datasets summarized in Table 1, UMAFall was selected because it provides IMU recordings from multiple body locations, including the wrist, waist, ankle, and chest, making it suitable for evaluating knowledge transfer from a multi-location teacher model to a wrist-only student model.

**Figure 1:**
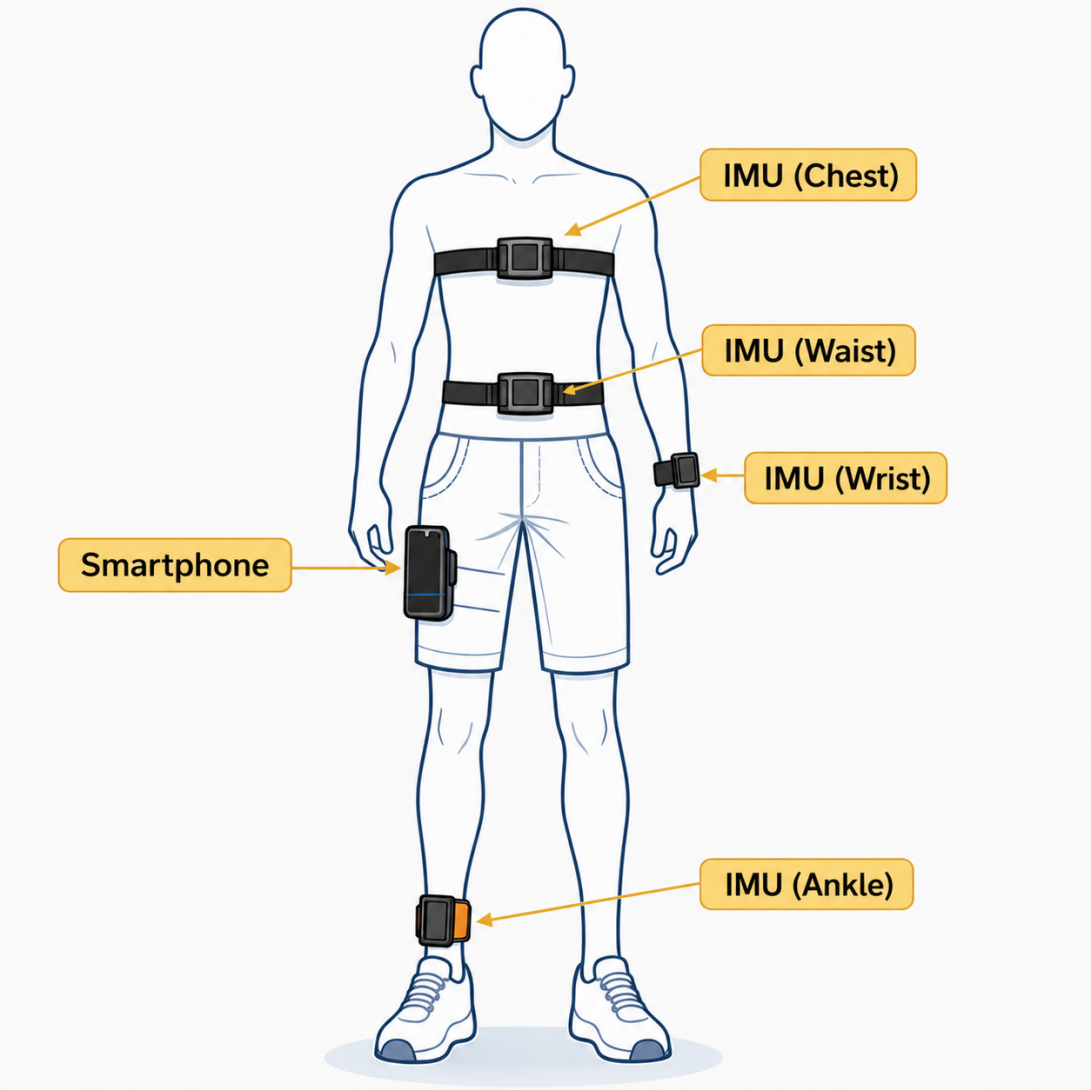
Sensor location for data collection in the UMAFall dataset [41].

The UMAFall dataset is a publicly available benchmark developed by the University of Málaga for human activity recognition and fall detection [41]. The original UMAFall publication presents data collected from 17 subjects, comprising 7 males and 10 females. However, the version of the dataset accessed from the official source and utilized in this study included records from 19 subjects. Consequently, Table 2 provides a summary of the dataset information based on the publicly available data. The dataset comprises 12 daily activities (e.g., walking and getting up from a chair) and 3 fall types: lateral, forward, and backward falls. Data were collected from multiple sensing locations, as shown in Figure 1 [41]. In this study, fall detection was formulated as a binary classification task, in which all activities of daily living were labeled as non-fall, and all fall activities were labeled as fall.

Although the UMAFall dataset has a limited number of participants, the Transformer teacher model was trained on trial-level temporal IMU windows rather than on subject-level observations. The external SensorTag IMU nodes in the UMAFall dataset were sampled at 20 Hz, and each activity trial lasted 15 s [41]; therefore, each trial provided an approximately 300-sample sequence representing the full temporal pattern of an ADL or fall event. This trial-level representation provided temporally rich inputs for the Transformer architecture, allowing the model to learn motion dynamics across the full duration of each activity. In addition, the binary formulation reduced the output-space complexity compared with multi-class activity recognition. Effective training of the Transformer teacher model was further supported by the use of multi-location IMU inputs, training-only data augmentation, and focal loss to address class imbalance.

### 2.2. Data Preprocessing

Data preprocessing involved standardizing data formats and removing corrupt or incomplete entries. This study used only accelerometer and gyroscope signals from the UMAFall IMU dataset. Prior to dataset creation, these channels were filtered using a fourth-order Butterworth low-pass filter with an 8 Hz cutoff to minimize high-frequency sensor noise. This preprocessing step was applied before subsequent dataset creation and model development [42,43]. After filtering, z-score normalization was applied to standardize the sensor channels. To avoid information leakage, normalization parameters were computed using only training data. Because the teacher and student models used different input configurations, normalization statistics were calculated separately for the multi-location teacher inputs and the wrist-only student inputs. Specifically, the channel-wise mean and standard deviation of the teacher training data were used to normalize the teacher inputs, whereas the channel-wise mean and standard deviation of the wrist-only student training data were used to normalize the student inputs. During validation, testing, and inference, the corresponding training-set mean and standard deviation were reused to transform each input stream, ensuring that unseen data were mapped to the same distribution as the training data.

**Figure 2:**
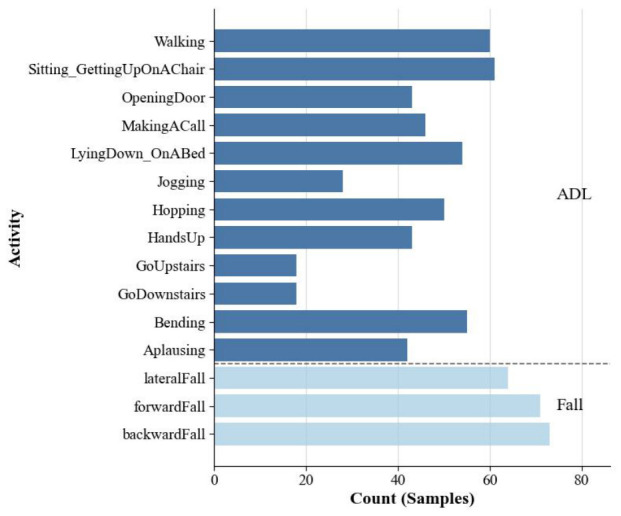
Distribution of activities among all trials and subjects (ADLs/falls).

**Figure 3:**
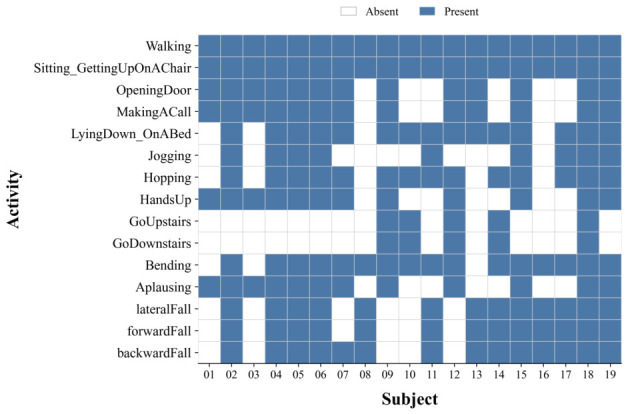
Distribution of activities (ADLs and falls) per subject.

The dataset distribution was then analyzed to identify missing or incomplete records, and a summary of the cleaned dataset is provided in Table 3. As shown in Figure 2, the dataset exhibits class imbalance, with the number of ADL samples exceeding fall events. In addition, Figure 3 shows that the downloaded dataset contained 19 subjects; however, Subjects 1, 3, 7, 9, 10, and 12 did not perform certain fall activities and were therefore excluded from the analysis. For the remaining subjects, data were available from all selected sensing locations.

### 2.3. Data Augmentation

To reduce overfitting caused by the limited dataset size, training-only data augmentation was applied during model training [44,45,46]. This approach is supported by recent wrist-based fall-detection research showing that data augmentation can improve model generalization when available training data are limited [45]. Augmentation was performed on the fly within the training data pipeline and was not applied to the validation or test data. Therefore, the number of original training windows remained unchanged, while augmented variations were generated dynamically during training.

For each training batch, augmentation was applied probabilistically to each sample with a probability of 0.35. When augmentation was applied, the IMU signals were randomly scaled by a factor between 0.95 and 1.05 and shifted by a value between −0.02 and 0.02. In addition, Gaussian noise with zero mean and a standard deviation of 0.002 was added to the signal. These transformations were used to simulate small variations in sensor amplitude, baseline offset, and measurement noise, which may improve robustness to minor differences in sensor recordings [47].

For the knowledge distillation model, the same random scaling and shifting factors were applied to the paired wrist-only student input and the corresponding multi-location teacher input to preserve consistency between paired samples. Gaussian noise was added separately to the student and teacher inputs. This augmentation strategy enriched the training process without modifying the validation or held-out test data.

### 2.4. Proposed Teacher–Student Framework

**Figure 4:**
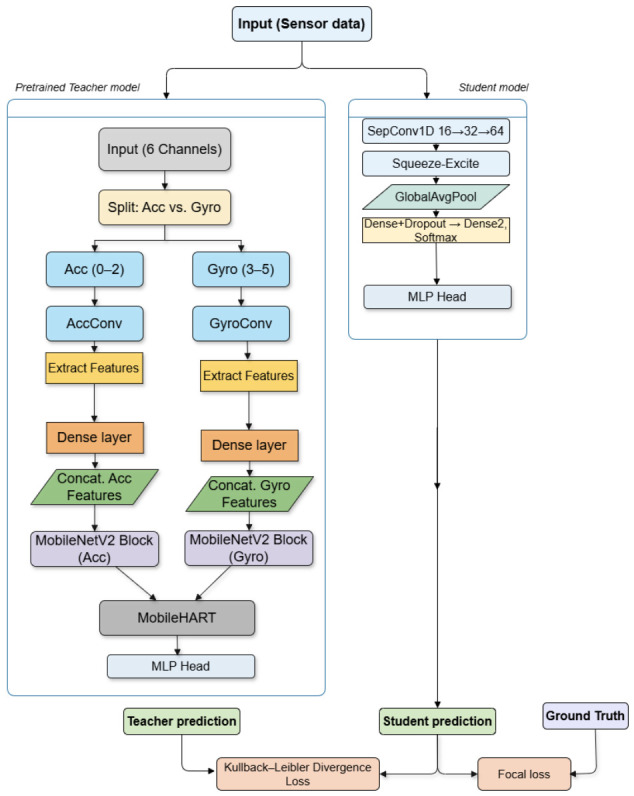
The schematic representation of the proposed teacher–student fall detection model.

The proposed teacher–student framework consisted of a Transformer-based teacher model and a lightweight CNN-based student model. The student model was trained using a hybrid objective that combined Kullback–Leibler divergence for soft teacher supervision with focal loss for the annotated class labels [38]. A schematic representation of the proposed framework is shown in Figure 4. Detailed descriptions of each model are provided in the following sections.

#### 2.4.1. Teacher Model

**Figure 5:**
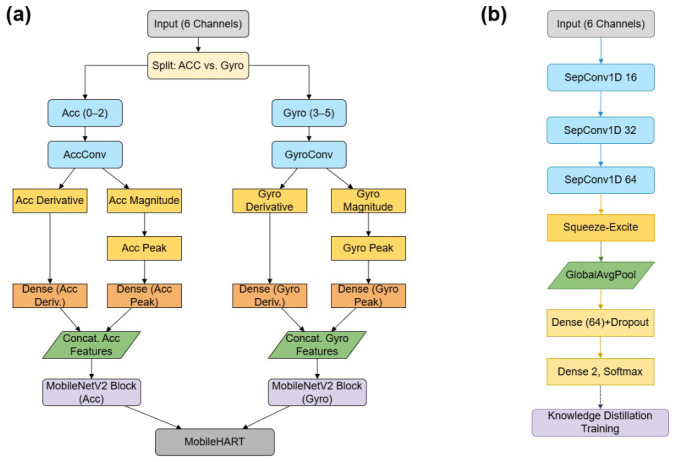
Structures of the proposed (**a**) teacher and (**b**) student models.

The proposed teacher model was built upon the MobileHARTXS framework [48], which is an optimized Transformer-based architecture with robust activity recognition capabilities. To enhance fall event detection, we added two extra branches specifically designed to extract features related to “sudden movement” for each sensor. These branches included derivative and peak-detection mechanisms that enhance the model’s ability to capture dynamic changes in sensor data. The extracted features were flattened and then passed through a fully connected (dense) layer with 64 units, followed by a Swish activation function. The output of the dense layer was passed to a convolutional encoder, which generated a feature vector per sensor, followed by the MV2 (MobileNet Version 2) block. The MV2 block improves computational efficiency while preserving important feature information by combining point-wise and depth-wise convolutions with residual connections, enabling efficient feature refinement and dimensional transformation with minimal computational overhead. The remaining components of the teacher model were structured similarly to the original MobileHART_XS architecture [48], with the addition of L2 regularization in the dense layers and the final multilayer perceptron (MLP) layer (Figure 5a).

#### 2.4.2. Focal Loss

Due to the class imbalance in the dataset, focal loss was used instead of the conventional cross-entropy loss. In fall detection datasets, the number of non-fall samples (majority class) typically exceeds the number of fall samples (minority class), which can bias models toward the majority class. Traditional cross-entropy loss treats all samples equally, often leading to reduced sensitivity with respect to rare but clinically important fall events. In contrast, focal loss dynamically down-weights majority-class examples and places greater emphasis on misclassified minority-class samples. This mechanism helps improve recall and F1 scores in imbalanced datasets, where reliable detection of rare but critical events such as falls is essential [49]. This was achieved through two mechanisms: (1) a modulating factor, which down-weights the contribution of easily classified majority-class samples, and (2) a weighting term α, which balances the relative importance of the minority and majority classes. As a result, focal loss effectively focuses the model’s learning on hard-to-classify and underrepresented fall instances while preventing the model from being overwhelmed by the frequent non-fall samples. The focal loss function based on the imbalance data weight (α vector) is defined below:

(1) $$\mathrm{Focal}\;\mathrm{loss}\left(p_{t}\right)=-\alpha {\left(1-p_{t}\right)}^{\gamma }\log\left(p_{t}\right)$$

where $p_{t}$ is the predicted probability assigned to the true class, α=[1.3,0.1] reweights fall versus non-fall classes, and γ=2 focuses learning on difficult examples.

#### 2.4.3. Student Model

To achieve an efficient, low-capacity network capable of approximating the complex decision boundary of the teacher model, a lightweight CNN-based student model was designed, as illustrated in Figure 5b. The student model received wrist-worn IMU data as input, consisting of three accelerometer and three gyroscope channels. The architecture included three separable one-dimensional convolutional layers. The first layer used 16 filters with a kernel size of 3 and stride of 1, followed by batch normalization. The second layer used 32 filters with a kernel size of 5 and stride of 2, followed by batch normalization. The third layer used 64 filters with a kernel size of 5 and stride of 2, followed by batch normalization. Swish activation was used in the convolutional layers.

After the convolutional feature extraction block, a squeeze-and-excite module was applied to recalibrate channel-wise feature responses. The resulting features were then passed through a global average pooling layer, followed by a dense layer with 64 units, dropout regularization, and a final dense output layer with two units corresponding to the fall and non-fall classes. This compact architecture contained approximately 12.3 thousand trainable parameters.

#### 2.4.4. Knowledge Distillation Framework

Because the student model is constrained to wrist-worn IMU data and has substantially fewer parameters than the multi-location teacher model, knowledge distillation was used to improve its ability to learn fall-related decision patterns from the teacher. In this framework, the teacher model was trained using IMU data from multiple body locations, including the chest, waist, ankle, and wrist, whereas the student model received only the wrist IMU signal from the same activity trial. Therefore, the teacher model served as a privileged-information model during training, providing supervision based on richer multi-location motion patterns that were not directly available to the student model.

Although the teacher and student models used different sensor inputs, output-logit distillation remained valid because both models predicted the same binary output space: fall and non-fall. Thus, the teacher’s softened output probabilities provided class-level supervisory information that guided the wrist-only student model toward the teacher’s decision behavior. The additional teacher sensors were used only during training to generate a stronger supervision signal and were not required during student-model inference. This strategy is consistent with wearable knowledge distillation studies in which a compact student model with fewer sensor modalities or sensor locations learns from a richer multi-modal or multi-position teacher model [39]. The proposed knowledge distillation procedure is summarized in Algorithm 1.

During training, the student model was optimized using a hybrid loss function composed of hard and soft loss terms. The hard loss was computed using focal loss based on the ground-truth fall and non-fall labels, helping the model focus on difficult and underrepresented fall samples. The hard loss was defined as follows:

(2) $$L_{\mathrm{hard}}=-\alpha {\left(1-p_{t}\right)}^{\gamma }\log\left(p_{t}\right)$$

where $p_{t}$ is the predicted probability assigned to the true class, α=[1.3,0.1] reweights fall versus non-fall classes, and γ=2 focuses learning on difficult examples.

The soft loss was computed using Kullback–Leibler divergence (KLD) between the softened output distributions of the teacher and student models. The Kullback–Leibler divergence was defined as follows [38]:

(3) $$D_{\mathrm{KL}}\left(p\|q\right)=\sum_{i}p_{i}\log\left(\frac{p_{i}}{q_{i}}\right)$$

where $p_{i}$ and $q_{i}$ denote the teacher and student probabilities for class *i*, respectively.
**Algorithm 1** Knowledge Distillation Framework for Wrist-Based Fall Detection1:**Input:**2:Multi-location IMU data $x_{T}$, wrist-only IMU data $x_{S}$, binary labels *y*, temperature *T*, and loss weight λ.3:**Output:**4:Trained wrist-only student model for Fall/Non-fall classification.5:**1. Teacher Model Training**6:Train the teacher model using multi-location IMU data $x_{T}$ and ground-truth labels *y*.7:Save the trained teacher weights and freeze the teacher model.8:**2. Paired Data Preparation**9:Pair each multi-location input $x_{T}$ with its corresponding wrist-only input $x_{S}$ from the same activity trial.10:Ensure that each paired input $\left(x_{T},x_{S}\right)$ shares the same binary label *y*.11:**3. Student Knowledge Distillation Training**12:Initialize the lightweight CNN student model.13:**For each training batch:**14:Compute teacher logits $t(x_{T})$ using the frozen teacher model.15:Compute student logits $s(x_{S})$ using the student model.16:Compute softened probabilities $p_{T}=\sigma \left(t\left(x_{T}\right)/T\right)$ and $q_{T}=\sigma \left(s\left(x_{S}\right)/T\right)$.17:Compute hard loss $L_{\mathrm{hard}}$ using focal loss and ground-truth labels *y*.18:Compute soft loss $L_{\mathrm{soft}}=T^{2}D_{\mathrm{KL}}\left(p_{T}\|q_{T}\right)$.19:Compute total loss $L_{\mathrm{total}}=\lambda L_{\mathrm{hard}}+\left(1-\lambda \right)L_{\mathrm{soft}}$.20:Update only the student model parameters.21:**End For**22:**4. Inference**23:Use only wrist IMU input $x_{S}$ for Fall/Non-fall prediction.

The soft distillation loss was then computed as follows [38]:

(4) $$L_{\mathrm{soft}}=T^{2}D_{\mathrm{KL}}\left(p_{T}\|q_{T}\right)$$

where

(5) $$p_{T}=\sigma \left(\frac{t(x_{T})}{T}\right),\;q_{T}=\sigma \left(\frac{s(x_{S})}{T}\right)$$

Here, $t(x_{T})$ and $s(x_{S})$ are the teacher and student model logits, respectively; $x_{T}$ represents the multi-location teacher input; $x_{S}$ represents the wrist-only student input; σ(·) denotes the softmax function. The parameter *T* is the distillation temperature used to soften the teacher and student probability distributions. When T>1, the softmax output becomes less peaked, allowing the student model to learn richer class-probability information from the teacher model rather than only the hard class label. The factor $T^{2}$ was included to compensate for the gradient scaling effect introduced by temperature-based softening. In this study, T=4.0, which was selected empirically based on trial-and-error experiments that produced the best validation performance.

The total training loss was defined as follows:

(6) $$L_{\mathrm{total}}=\lambda L_{\mathrm{hard}}+\left(1-\lambda \right)L_{\mathrm{soft}}$$

where $L_{\mathrm{hard}}$ is the focal loss computed from the ground-truth labels, $L_{\mathrm{soft}}$ is the knowledge distillation loss, and λ controls the relative contribution of the hard and soft losses. In this study, λ=0.6.

During knowledge distillation, the student model’s weights were optimized using the AdamW optimizer [50], while the teacher model remained frozen during training. Compared to the standard Adam optimizer, AdamW decouples weight decay from the gradient-based update rule, which prevents unintended interactions between L2 regularization and adaptive learning rates. This modification leads to better generalization and improved convergence stability, especially in deep neural networks trained on relatively small or imbalanced datasets [50].

### 2.5. Model Training and Validation

All model training and evaluation were conducted using Python 3.8 on a Windows 11 system equipped with an Intel Core i9 CPU, 32 GB of RAM, and an NVIDIA RTX 4090 GPU. For each sensing location, IMU data were represented as fixed-length windows with 300 time steps and 6 sensor channels, corresponding to three accelerometer and three gyroscope axes. The student model used only the wrist IMU window, whereas the teacher model used the corresponding synchronized windows from four sensing locations: waist, wrist, ankle, and chest. For model comparison, data from Subjects 2 and 5 were held out as an independent test set, while the remaining data were randomly divided into training and validation subsets using a 70/30 split. This splitting procedure was applied consistently across the Transformer-based teacher model, the independent CNN student model, and the knowledge-distilled student model.

For the Transformer-based teacher model, the AdamW optimizer was used with an initial learning rate of $1\times 10^{-4}$, a batch size of 256, and a dropout rate of 0.3. The projection dimension was set to 96, the number of attention heads was set to 4, and the convolution kernel sizes were set to [3, 7, 15, 31, 31, 31]. The patch length and time step were both set to 16.

The lightweight CNN student model was trained using the same batch size of 256. The student model included three separable one-dimensional convolutional layers with 16, 32, and 64 filters and kernel sizes of 3, 5, and 5, respectively. L2 regularization was applied to improve generalization. During knowledge distillation, the temperature parameter was set to T=4.0, the hard-loss weighting coefficient was set to λ=0.6, focal-loss γ was set to 2.0, and the focal-loss class weights were set to [1.3, 0.1] for fall and non-fall, respectively. Both the teacher and student models were trained for 50 epochs.

### 2.6. Performance Evaluation Metrics

The performance of all models was evaluated using standard evaluation metrics, including accuracy, precision, recall, and the F1 score [11,35]. These metrics are defined based on true positives (TPs), true negatives (TNs), false positives (FPs), and false negatives (FNs). TP represents correctly detected fall events, TN denotes correctly identified non-fall activities, FP corresponds to non-fall activities incorrectly classified as falls, and FN indicates fall events that were not detected by the model. The evaluation metrics are defined as follows:

(7) $$\mathrm{Accuracy}=\frac{TP+TN}{TP+FN+TN+FP}$$

(8) $$\mathrm{Precision}=\frac{TP}{TP+FP}$$

(9) $$\mathrm{Recall}=\frac{TP}{TP+FN}$$

(10) $$F1\text{-}\mathrm{score}=\frac{2\times \mathrm{Precision}\times \mathrm{Recall}}{\mathrm{Precision}+\mathrm{Recall}}$$

Accuracy provides an overall measure of correct classifications, precision reflects the proportion of detected falls that were correctly identified, recall measures the model’s ability to detect actual fall events, and the F1 score balances precision and recall, providing a single robust indicator of performance. To evaluate whether focal loss improves the model’s sensitivity to fall events compared with conventional cross-entropy, the Transformer-based teacher model was trained using both loss functions. Finally, the performance of the lightweight CNN-based student model was evaluated under two conditions: (1) when trained using the proposed knowledge distillation framework and (2) when trained independently from the teacher model.

### 2.7. Computational Complexity and Inference-Time Analysis

To compare the computational efficiency of the teacher and student models, three complementary measures were used: the number of trainable parameters, the computational complexity in mega floating-point operations (MFLOPs), and the average inference time per input window. The number of trainable parameters was obtained from the model summary and reflects the memory and storage requirement of each model. Because the teacher model used IMU data from four sensing locations, its input window had a shape of 300×24, corresponding to 300 time steps and 24 channels, including six channels collected from each sensing location. In contrast, the student model used only the wrist IMU signal and therefore had an input window of 300×6, corresponding to 300 time steps and six channels from one sensing location.

The MFLOPs were computed from the corresponding model computation graph for each input configuration. Following the convention used in [51], the convolutional computational cost was counted in a multiply–accumulate style. For a two-dimensional convolution, the computational cost is defined as

(11) $${\mathrm{FLOPs}}_{\mathrm{conv}}=D_{k}\times D_{k}\times D_{i}\times W_{o}\times H_{o}\times N$$

where $D_{k}$ is the kernel size, $D_{i}$ is the number of input channels, $W_{o}$ and $H_{o}$ are the output dimensions, and *N* is the number of output filters.

For one-dimensional time-series convolution, this formulation reduces to

(12) $${\mathrm{FLOPs}}_{\mathrm{conv}1D}=K\times C_{\mathrm{in}}\times L_{\mathrm{out}}\times C_{\mathrm{out}}$$

where *K* is the temporal kernel length, $C_{\mathrm{in}}$ is the number of input channels, $L_{\mathrm{out}}$ is the output sequence length, and $C_{\mathrm{out}}$ is the number of output channels.

For depthwise-separable one-dimensional convolution, the computational cost was computed as the sum of the depthwise and pointwise components:

(13) $${\mathrm{FLOPs}}_{\mathrm{sepconv}1D}=K\times C_{\mathrm{in}}\times L_{\mathrm{out}}+C_{\mathrm{in}}\times C_{\mathrm{out}}\times L_{\mathrm{out}}.$$

Fully connected layers were counted as

(14) $${\mathrm{FLOPs}}_{\mathrm{dense}}=C_{\mathrm{in}}\times C_{\mathrm{out}}.$$

The total computational complexity of each model was obtained by summing the FLOPs over all computational layers and dividing by $10^{6}$ to report MFLOPs:

(15) $$\mathrm{MFLOPs}=\frac{\sum_{l}{\mathrm{FLOPs}}_{l}}{10^{6}},$$

where *l* indexes the computational layers of the model. Table 4 summarizes the parameters used in the FLOP calculation.

The inference time was measured using the trained model weights on the held-out test data. For the teacher model, inference time was measured using the full multi-location input window with dimensions 300×24, corresponding to 300 time steps and 24 channels from four sensing locations. For the student models, inference time was measured using the wrist-only input window with dimensions 300×6, corresponding to 300 time steps and six channels from one sensing location.

For each model, several warm-up batches were first passed through the network to reduce graph-building and initialization effects. Inference time was then measured over multiple batches using wall-clock execution time. For a given model, the average inference time per input window was calculated as follows:

(16) $$t_{\mathrm{window}}=\frac{\sum_{b=1}^{B}t_{b}}{\sum_{b=1}^{B}n_{b}}$$

where *B* is the number of measured batches, $t_{b}$ is the inference time for batch *b*, and $n_{b}$ is the number of input windows in batch *b*.

Because inference time was measured directly using each model’s actual input configuration, no additional scaling factor was applied. Thus, the teacher-model inference time represents the time required to process one complete multi-location input window, whereas the student-model inference time represents the time required to process one wrist-only input window.

## 3. Results and Discussion

### 3.1. Effect of Focal Loss on Teacher Model Performance

Table 5 presents the performance of the Transformer-based teacher model trained with cross-entropy and focal loss. The evaluation was performed using Subjects 02 and 05, which were reserved for testing and were not included in the training set. Replacing cross-entropy with focal loss improved the model’s ability to detect falls, increasing recall from 90% to 96.7% while maintaining high overall accuracy. The F1 score also increased from 93.1% to 96.7%. These results indicate that focal loss improved the model’s sensitivity to fall events without substantially reducing its ability to recognize non-fall activities.

This improvement is consistent with the class-imbalance problem commonly observed in fall detection. Falls are rare and short-duration events, whereas activities of daily living (ADLs) occur more frequently and for longer periods [32,52,53]. As summarized in Table 2, the dataset used in this study contained fewer fall events than ADL activities, resulting in an imbalanced training distribution. Under this condition, standard cross-entropy can bias the model toward the majority non-fall class. Focal loss addresses this issue by assigning greater importance to difficult and underrepresented samples, which helps the model focus on fall events. The recall improvement in Table 5 therefore supports the use of focal loss for training the teacher model.

### 3.2. Effect of Knowledge Distillation on Wrist-Only Student Model Performance

After training the teacher model, its knowledge was transferred to a compact wrist-only student model through knowledge distillation. Table 6 compares the teacher model, the independently trained CNN, and the student model trained with knowledge distillation (KD-CNN). The comparison focuses on the test data used for the detailed model evaluation and allows the effect of teacher-guided training to be examined under the same wrist-only input condition for the two student models.

The teacher model achieved the highest overall performance, with 97.6% accuracy, 96.7% precision, 96.7% recall, and an F1 score of 96.7%. This result is expected because the teacher model used information from multiple sensing locations, which provides a more complete representation of body motion during falls and ADLs. In contrast, the independently trained CNN relied only on wrist-worn IMU data and achieved 90.2% accuracy, 84.4% precision, 90.0% recall, and an F1 score of 87.1%. The lower precision and F1 score indicate that wrist-only training with hard labels alone was less effective in separating fall events from non-fall activities.

KD-CNN improved the performance of the wrist-only student model, achieving 95.1% accuracy, 93.3% precision, 93.3% recall, and an F1 score of 93.3%. These improvements suggest that the teacher model provided useful supervisory information during training. Because the teacher model learned from multiple sensing locations, its output probabilities contained class-separation information that was not available from the wrist signal alone. Through knowledge distillation, this information helped the compact wrist-only student model learn a more effective decision boundary.

**Figure 6:**
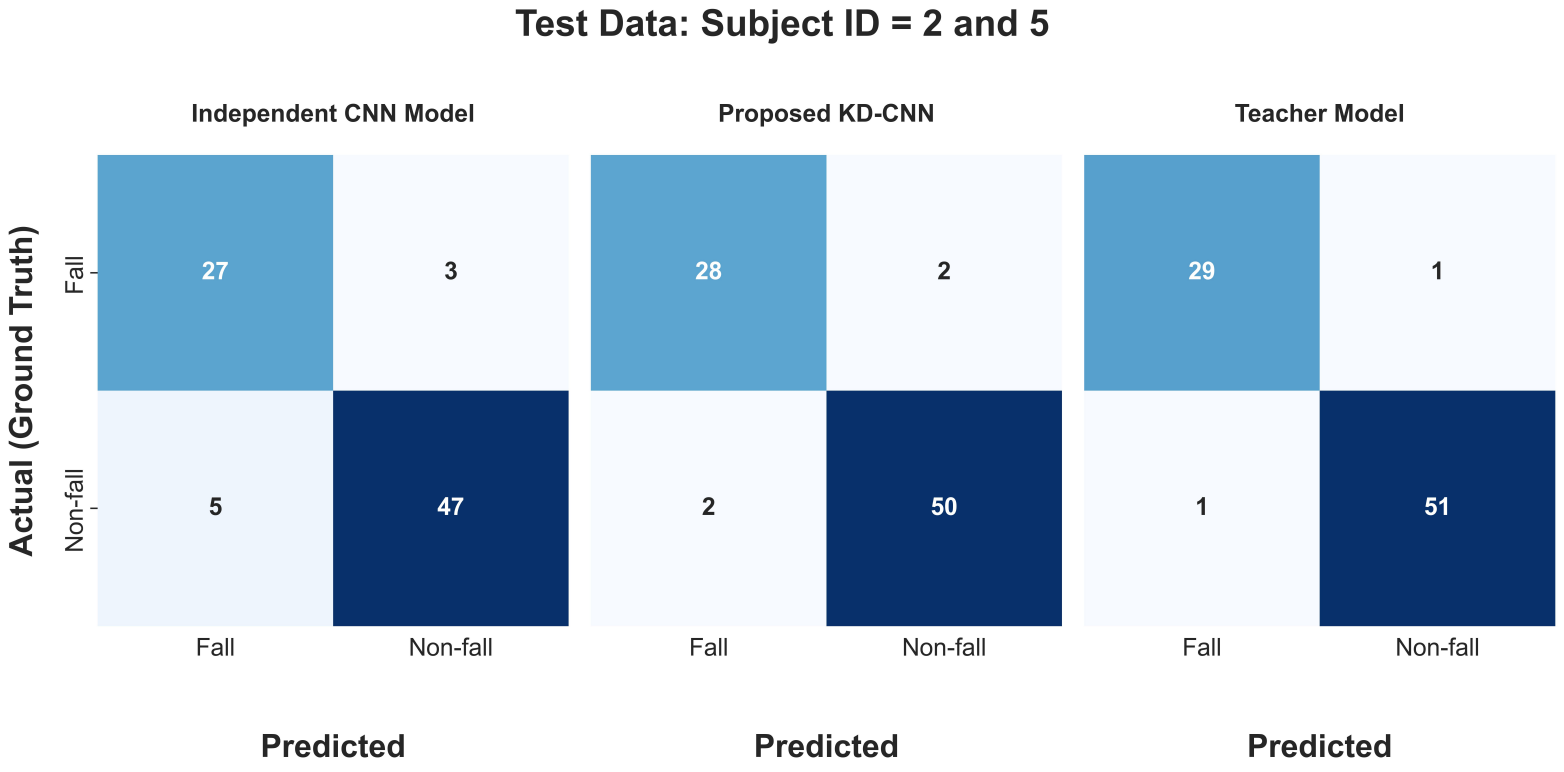
Comparison of independent CNN, proposed KD-CNN, and teacher models.

The raw confusion matrices for the independent CNN, KD-CNN, and teacher model are shown in Figure 6. Subjects 2 and 5 were excluded from the training process and did not contribute to the training dataset. As a result, there was no subject-level overlap between the training and testing sets. The confusion matrix of the KD-CNN supports the results in Table 6, showing improved fall classification compared with the independently trained CNN while maintaining performance close to the teacher model.

### 3.3. Leave-One-Subject-Out Cross-Validation Analysis

In addition to the detailed held-out test evaluation, leave-one-subject-out (LOSO) cross-validation was conducted as a supplementary subject-level robustness analysis. The primary objective of this study was to evaluate the feasibility of transferring knowledge from a multi-location teacher model to a compact wrist-only student model rather than to develop a fully optimized subject-independent fall-detection system. Therefore, the fixed held-out test evaluation was used as the main comparison because it allowed the independently trained CNN and the KD-CNN to be evaluated under identical data splits, architectures, preprocessing procedures, and hyperparameter settings. LOSO cross-validation was included to examine whether the relative benefit of knowledge distillation was preserved when different subjects were held out.

**Figure 7:**
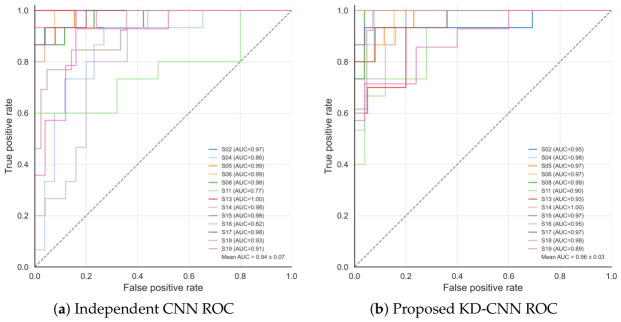
Leave-one-subject-out cross-validation results. (**a**) ROC curves of the independently trained CNN. (**b**) ROC curves of the student model trained with knowledge distillation (KD-CNN). Each fold corresponds to one held-out subject from the valid subjects described in Section 2.2. **Note:** The dotted lines represent the random-classifier baseline.

As shown in Figure 7, each valid subject described in Section 2.2 was used once as the held-out test subject. Thus, each fold corresponds to one subject; for example, Fold 1 corresponds to Subject 02, Fold 2 corresponds to Subject 04, and the sequence continues through Fold 13, which corresponds to Subject 19. Because several hyperparameters, including the focal-loss class weights and distillation parameters, were selected based on the training-data distribution and validation performance, the LOSO results should be interpreted as a supplementary feasibility analysis rather than as definitive evidence of fully optimized subject-independent generalization.

The improvement observed in Table 6 was also reflected in the LOSO results shown in Figure 7. Figure 7a,b show the ROC curves for the independently trained CNN and the KD-CNN, respectively. Across the subject-level folds, KD-CNN achieved a higher and more stable AUC than the independently trained CNN. The independently trained CNN obtained an average AUC of 0.94±0.07, whereas the KD-CNN achieved an average AUC of 0.96±0.03. This suggests that knowledge distillation improved the ability of the wrist-only model to separate fall and non-fall samples across held-out subjects, although further work is needed to optimize hyperparameters specifically for subject-independent deployment.

One reason for the lower and more variable AUC of the independently trained CNN may be the similarity between fall events and ADLs involving sudden wrist movements. Activities such as “Hands up” and “Applauding” can produce rapid wrist acceleration patterns that resemble fall-related motion when only wrist IMU data are available. As a result, the independently trained CNN may assign higher fall probabilities to some fall-like ADLs, reducing the separation between fall and non-fall classes. The KD-CNN was less affected by this issue because teacher-guided training provided additional information learned from multi-location sensing, helping the student model better distinguish true falls from sudden non-fall wrist movements.

To further assess the stability of the main held-out test findings, both the independently trained CNN and the KD-CNN were trained and evaluated across five independent iterations on Subjects 2 and 5 using different random initializations. The same model architecture, batch size, learning rate, number of epochs, and other hyperparameter settings were kept fixed across runs; only the random seed was changed. Across these runs, KD-CNN achieved an accuracy of 94.17%±1.85% and a fall F1 score of 91.92%±2.39%. In comparison, the independently trained CNN achieved an accuracy of 89.32%±2.15% and a fall F1 score of 88.50%±4.20%. These results show that the KD-CNN maintained higher mean performance and lower variations in fall F1 scores across random initializations, further supporting the stability of the proposed knowledge distillation framework under the primary evaluation setting.

### 3.4. Comparison with Existing Wrist-Based Fall Detection Methods

To place the proposed KD-CNN in the context of prior wrist-based fall detection studies, Table 7 compares its performance with existing methods evaluated on the UMAFall dataset. These comparisons should be interpreted cautiously because the studies may differ in preprocessing procedures, subject-selection criteria, train–test splitting strategies, evaluation protocols, and metric definitions. In addition, to the best of our knowledge, prior fall-detection studies using knowledge distillation have mainly focused on reducing model complexity under similar sensing configurations. Therefore, a direct methodological comparison with an equivalent teacher–student framework was not available, and Table 7 is intended to provide a contextual reference rather than a strict direct ranking of methods.

Within this context, the proposed KD-CNN achieved competitive fall-detection performance while using a substantially smaller model architecture than previously reported deep learning approaches. The student model contained approximately 12.3 k trainable parameters compared with 136,321 parameters for the CNN model in [54]. Under the evaluation protocol used in the present study, the proposed KD-CNN achieved recall and F1 score values that were within the range of previously reported UMAFall wrist-based methods while using fewer trainable parameters. This result suggests that the proposed model may provide a favorable balance between compactness and fall-event sensitivity, which is important for wrist-based fall detection where missed falls are particularly undesirable.

However, because the compared studies were not evaluated under a unified experimental protocol, the results should not be interpreted as definitive evidence of superiority over prior methods. Rather, the comparison indicates that the proposed knowledge-distilled student model can achieve performance within the range of existing UMAFall wrist-based methods while requiring substantially fewer trainable parameters. These findings support the feasibility of using knowledge distillation to transfer useful information from a multi-location teacher model to a compact wrist-only student model, while highlighting the need for future benchmarking under standardized subject splits and preprocessing procedures.

### 3.5. Limitations and Future Directions

This study has several limitations. First, the UMAFall dataset used in this study includes participants younger than 55 years and does not include elderly adults. Therefore, the results may not fully represent fall patterns, gait characteristics, reaction movements, or wrist-motion behavior in older adults, who are the main target population for fall detection systems. Future research should evaluate the proposed model using data collected from elderly participants.

Second, although the proposed model was designed for wrist-worn sensing, it was not tested on an actual smartwatch. Therefore, latency, memory use, battery consumption, and robustness during routine smartwatch use were not evaluated. Future work should validate the model on wearable hardware under free-living conditions.

Third, the dataset contains a limited range of activities of daily living and does not include sufficient near-fall or fall-like activities with motions similar to falls, such as zipping up clothing, eating, reaching, or other rapid arm movements. This may limit the ability to assess false alarms in realistic daily use. Future studies should include a larger and more diverse dataset with older adults, realistic daily activities, near-fall events, fall-like wrist movements, and natural variations in movement speed and arm motion. Techniques such as calibration, domain adaptation, and model personalization may further improve performance across different users and deployment conditions.

## 4. Conclusions

This study developed a knowledge distillation framework to train an accurate and lightweight model for fall detection using wrist-worn IMU data. A Transformer-based teacher model was first trained with focal loss to improve fall-event learning under class imbalance. The knowledge learned by the teacher model was then transferred to a compact CNN-based student model that used only wrist-worn IMU signals. The results showed that knowledge distillation improved the student model compared with an independently trained CNN under the same evaluation setting.

The findings indicate that teacher-guided training can help a wrist-only CNN learn more discriminative fall-related patterns, while maintaining a smaller architecture than the teacher model. This supports the feasibility of using knowledge distillation to reduce model complexity for wrist-based fall detection. However, the proposed model should be interpreted as a compact and promising wrist-only approach rather than a fully validated smartwatch deployment system.

Several limitations remain. The dataset did not include elderly participants, although older adults are the main target population for fall detection systems. In addition, the dataset contained a limited range of near-fall and fall-like daily activities, such as eating, zipping up clothing, reaching, and other rapid wrist movements that may resemble falls. The model was also not evaluated on an actual smartwatch, so real-time latency, memory use, and power consumption were not assessed. Future work should evaluate the model on larger and more diverse datasets that include elderly participants, realistic near-fall activities, and free-living smartwatch data. Hardware-level validation is also needed to determine whether the proposed framework can be reliably deployed in practical wearable fall detection systems.

## Figures and Tables

**Table 1:** Summary of public wearable-sensor datasets and sensing-point configurations [40].

Dataset	Number of Sensing Points	Recorded Magnitudes	Position of the Sensing Points
DLR	1	A, G, M	Waist
MobiFall & Mobile Act	1	A, G, O	Thigh (trouser pocket)
TST Fall Detection	2	A	Waist; right wrist
tFall	1	A	Alternatively thigh (right or left pocket) or handbag (left or right side)
UR Fall Detection	1	A	Waist (near the pelvis)
Cogent Labs	2	A, G	Chest; thigh
Gravity Project	2	A	Thigh (smartphone in a pocket); wrist (smartwatch)
Graz	1	A, O	Waist
UMAFall	5	A, G, M	Ankle; chest; thigh (right trouser pocket); waist; wrist
SisFall	1	A, A, G	Waist
UniMiB SHAR	1	A	Thigh (left or right trouser pocket)

A: Acceleration; G: gyroscope; O: orientation measurement; M: magnetometer.

**Table 2:** Summary of the UMAFall dataset characteristics before cleaning.

Dataset Source	Subjects	Age	Weight	Samples (ADLs/Falls)	Duration	Type of ADL	Type of Fall
Downloaded dataset	19	18–55	50–93	746 (538/208)	15 s	Walking; sitting; getting up from a chair; opening a door; making a call; lying on a bed; jogging; hopping; hands up; going upstairs; going downstairs; bending; applauding	Lateral fall; forward fall; backward fall

**Table 3:** Summary of the UMAFall dataset after cleaning.

Number of Subjects	Samples (ADLs/Falls)	Duration	Type of ADL	Type of Fall
13	590 (388/202)	15 s	Walking; sitting; getting up from a chair; opening a door; making a call; lying on a bed; jogging; hopping; hands up; going upstairs; going downstairs; bending; applauding	Lateral fall; forward fall; backward fall

**Table 4:** Definition of parameters used for MFLOP calculation.

Symbol	Definition
$D_{k}$	Kernel size for two-dimensional convolution
$D_{i}$	Number of input channels for two-dimensional convolution
$W_{o}$	Output width of the convolution layer
$H_{o}$	Output height of the convolution layer
*N*	Number of output filters
*K*	Temporal kernel length for one-dimensional convolution
$C_{\mathrm{in}}$	Number of input channels
$C_{\mathrm{out}}$	Number of output channels or filters
$L_{\mathrm{out}}$	Output sequence length after one-dimensional convolution
*l*	Index of a computational layer in the model
${\mathrm{FLOPs}}_{l}$	Floating-point operations of layer *l*

**Table 5:** Comparison of the teacher model’s performance using focal loss and cross-entropy loss functions.

	Performance Metrics			
Loss Function	Precision (%)	Recall (%)	Accuracy (%)	F1 Score (%)
Cross-entropy loss	96.4	90	95.1	93.1
Focal loss	96.7	96.7	97.6	96.7

**Table 6:** Comparison of the performance and computational efficiency of the student model trained based on knowledge distillation, a similar model trained independently of the teacher model, and the teacher model.

	Performance and Efficiency Metrics						Number of Trainable Parameters
Model	Precision (%)	Recall (%)	Accuracy (%)	F1 Score (%)	Inference Time (ms)	MFLOPs	
Independent CNN model	84.4	90.0	90.2	87.1	∼0.23	0.8294	∼12.3 k
Proposed KD-CNN	93.3	93.3	95.1	93.3	∼0.23	0.8294	∼12.3 k
Teacher model	96.7	96.7	97.6	96.7	∼2.37	39.0883	∼1.3 M

**Table 7:** Comparison of wrist-based fall detection methods on the UMAFall dataset.

Method	Precision (%)	Recall (%)	F1 Score (%)	Trainable Parameters
CNN (2022) [54]	**97.61**	87.63	92.35	136,321
Threshold (2021) [55]	84.93	86.70	85.81	–
PerF (2016) [41]	91.40	75.60	82.75	–
Proposed KD-CNN	93.3	**93.3**	**93.3**	∼**12,300**

**Note:** Bold values indicate the best result in each category: the highest value for Precision, Recall, and F1 Score, and the lowest value for Trainable Parameters.

## Data Availability

The data analyzed in this study are publicly available on Figshare at https://figshare.com/articles/dataset/UMA_ADL_FALL_Dataset_zip/4214283 (accessed on 10 December 2025). The UMAFall dataset was originally published by Casilari, Santoyo-Ramón, and Cano-García [41].
